# Onset of ADL and IADL limitation among Chinese middle-aged and older adults

**DOI:** 10.1371/journal.pone.0287856

**Published:** 2023-07-17

**Authors:** Wenyuan Zheng, Zhiyong Huang

**Affiliations:** 1 Department of Insurance, School of Finance, Southwestern University of Finance and Economics, Chengdu, China; 2 Department of Social Security, School of Public Administration, Southwestern University of Finance and Economics, Chengdu, China; Instituto Nacional de Geriatria, MEXICO

## Abstract

It is important to measure the prevalence and onset of limitations for older adults to take critical day-to-day activities in the population. However, too often, only very older people are covered, and too few activities are included in the studies. Using a nationally representative sample from 2011 to 2018 (*N* = 16, 381), this study characterizes the limitation pattern covering ADL and IADL activities among middle-aged and older adults in China. We use survival models to characterize the limitation transition. We find that half of the population become limited in activities including housekeeping, toileting, managing money, and cooking in their early 70s, followed by shopping, bathing, transferring and dressing in their late 70s, continence, and taking medications in their early 80s, and feeding in their early 90s. In addition, women show significantly younger age of limitation onsets for all activities except continence.

## Introduction

The world population is aging. The number of adults aged 60 and above was around 1 billion worldwide in 2020, and this figure is expected to double, up to nearly 2.1 billion in 2050 [1]. According to the statistics of the United Nations, China has already had the largest population of older adults. Approximately 172 million older people aged 65 and above in 2020, accounting for 11.96% of its people. The figure is projected to reach 366 million by 2050, which equates to 26.07% of the national population [2]. Unprecedented population aging generates tremendous pressure on individuals, households, and society [3–5]. Under the context of population aging, World Health Organization (WHO) advocates healthy aging [1] to promote the well-being of older adults, mitigate inequities among different social groups, and ensure a sustainable and cost-effective care system. To achieve healthy aging for the society, it is necessary to maintain the functional ability and, therefore the independence and quality of life of people in old age. Before making any public policy or medical intervention regarding maintaining the function ability, it is crucial to measure the limitation for older adults to take critical day-to-day activities in the population [6].

Activity limitation is a crucial aspect of disability and functioning. The Disablement Model [7] provides a theoretical framework for understanding disability development, delineating three levels of disability: impairments, functional limitations, and disability. Impairments refer to abnormalities or losses in the body or body parts, while functional limitations pertain to performance at the individual level, and disability encompasses limitations in the performance of social roles. Recognizing that everyone experiences some degree of disability, the World Health Organization (WHO) developed the Biopsychosocial Model, which serves as the foundation for the International Classification of Functioning, Disability, and Health (ICF) [8]. The ICF replaces functional limitation and disability with positive terms like activity and participation, incorporating three levels of human functioning: body function, activity, and participation. Disability is viewed as malfunctioning at one or more of these levels: impairments, activity limitations, and participation restrictions. All forms of functioning are considered to result from the interplay between health conditions and contextual factors. Personal factors such as age, gender, and behaviors, as well as environmental factors like social support and architectural characteristics, are among the contextual factors.

Among measures of activity limitation, Activities of Daily Living (ADL) [9] and Instrumental Activities of Daily Living (IADL) [10] are commonly utilized tools in large-scale surveys and clinical screenings [11–14]. ADLs encompass basic personal care activities, while IADLs involve activities necessary for independent living within a community. Studies focusing on ADL activities have found that individuals often experience a decline in abilities related to lower extremity strength first, followed by upper extremity limitations [15, 16]. However, the order of onset can be influenced by living facilities and arrangements, which vary greatly across countries [17]. Sex differences exist in the risk of developing limitations in ADL and IADL activities. It has been documented that women face a higher risk of limitation and tend to experience limitations at an earlier age than men in most activities [15, 18]. Previous studies have also suggested that combining ADL and IADL items is likely to enhance measurement sensitivity by encompassing a broader range of activities [19]. Nevertheless, the evidence regarding the hierarchical order of IADL and ADL limitations is mixed [19–23].

Despite a substantial body of literature on activity limitation in aging, most studies have only included ADL items, focused on very old adults, or employed small-sized community-based samples from high-income countries. To the best of our knowledge, no studies have investigated the pattern of limitations encompassing ADL and IADL activities among middle-aged and older Chinese adults. Considering the distinct personal and environmental factors that Chinese older adults encounter, in line with the Disablement Model and Biopsychosocial Model, it is likely that they follow a different trajectory of disability development compared to their counterparts in high-income countries. For instance, China lacks disability-friendly living facilities, thereby making activities like transfers and toileting more challenging and potentially accelerating disability progression.

The primary objective of this study is to understand the pattern of activity limitation in China. In particular, this study characterizes the prevalence and onset of activity limitations measured by ADL and IADL items among both middle-aged and older adults in China, by utilizing a nationally representative survey consisting of 16,381 participants and spanning seven years, from 2011 to 2018. The hypotheses tested are:

(H1) Chinese older adults experience a lack of disability-friendly living facilities.

(H2) The onset of limitation in ADL and IADL activities is influenced by both health conditions and contextual factors, such as the availability of appropriate living facilities.

(H3) The absence of crucial facilities, such as toilets and showers, can accelerate the onset of ADL activity limitations, particularly in activities like toileting and bathing. Consequently, the onset of limitations in ADL and IADL activities may not follow a strict hierarchical order.

(H4) The timing and order of limitations in Chinese older adults differ from those observed in older adults from UK with superior living facilities, indicating the impact of disparities in facility provision on the development of activity limitations.

The findings of our study add to the existing literature in the following ways. First, previous studies have mainly focused on the high-income countries [15, 19–21, 23, 24], while evidence on the developing countries is recognized to be very sparse [25]. Second, most existing studies have relied on community-based samples, which are relatively small in size and cross-sectional [22]. In contrast, our study uses a nationally representative sample that is large in size and longitudinal in design. Last but not least, most studies include only the oldest-old adults as the target population and only ADL items as measures of activity limitation [15–17, 26], while we examine both middle-aged and older adults and investigate IADL items in addition to ADL items. It is pertinent to include middle-aged in the study of development of activity limitation in countries like China, where people can become limited in functional ability earlier than in developed countries. Moreover, including items beyond ADL enables us to evaluate a broader spectrum of activities likely to affect older people’s well-being and care needs.

## Methods

### Participants

We use the China Health and Retirement Longitudinal Study (CHARLS) data from 2011 to 2018. As a sister survey to the Health and Retirement Study (HRS), the English Longitudinal Study of Ageing (ELSA), and the Survey of Health, Ageing and Retirement in Europe (SHARE), CHARLS is designed as a nationally representative longitudinal survey for Chinese people who are aged 45 years or older. CRHALS collects a wide range of information on participants’ social, economic, and health circumstances. In the baseline survey conducted between June 2011 and March 2012, 17,708 participants were interviewed. Three follow-up surveys were fielded in 2013, 2015 and 2018, without counting the 2016 Life History Survey, which was drawn from the same population but for a particular purpose. Peking University IRB has approved the data collection (approval number IRB0000105211015). Written informed consents were obtained from all respondents. Full details of the survey can be found elsewhere [14].

**Fig 1:**
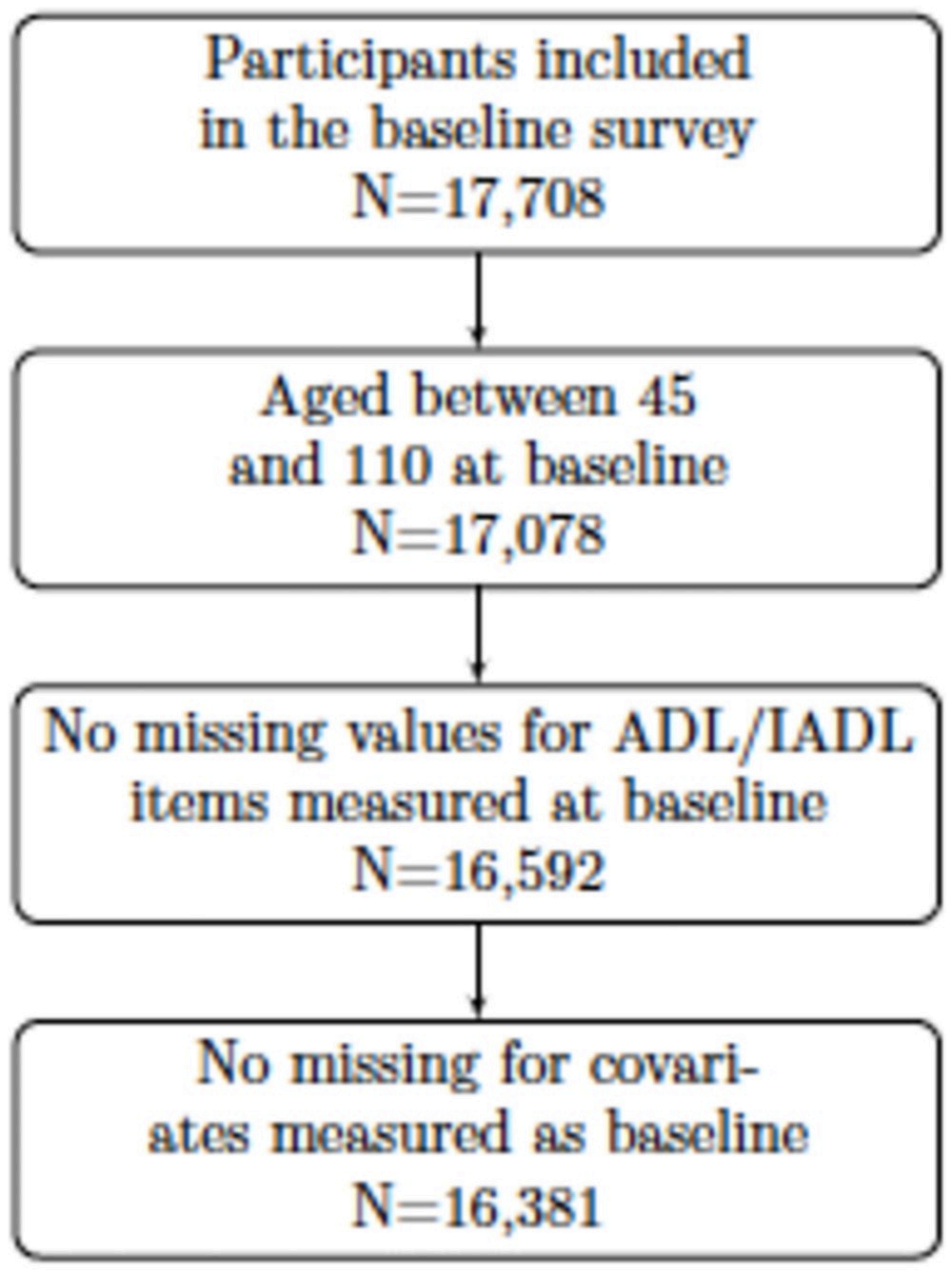
Study sample.

For this study, we have included participants who were interviewed and aged between 45 and 110 during the baseline survey, resulting in the exclusion of 630 individuals. Additionally, individuals with missing values in ADL/IADL activities (486 individuals) or covariates (211 individuals) during the baseline survey were also excluded. As a result, the final study sample consisted of 16,381 individuals (refer to Fig 1). Furthermore, when using survival analysis to estimate the time from no limitation to limitation for a specific ADL/IADL activity, we further restricted the sample of interest to participants who had no limitation in that particular activity at the time of entry into the survey.

### Measures

In CHARLS, limitations in activities are measured as self-reports on a wide range of basic and instrumental activities of daily living (Table 1 lists ADL/IADL questions included in the CHARLS questionnaire). Basic activities of daily living are assessed by the Katz index of ADLs [9], which comprise six domains: dressing, bathing, feeding, transferring, toileting and continence. Instrumental activities of daily living are adapted from Lawton and Brody IADL inventory [10]. Original Lawton and Brody scale includes using the phone, shopping, cooking, housekeeping, doing laundry, using transportation, taking medications, and managing money. CHARLS keeps activities in housekeeping, cooking, shopping, managing money and taking medications, and excludes questions on using the phone, doing laundry, and using transportation, which are considered less prevalent for Chinese older adults, especially those living in rural areas.

**Table 1:** ADL/IADL items in CHARLS.

Item	Survey question
	Because of health and memory problems,
Dressing	do you have any difficulty with dressing?
Bathing	do you have any difficulty with bathing or showering?
Feeding	do you have any difficulty with eating, such as cutting up your food?
Transferring	do you have any difficulty with getting into or out of bed?
Toileting	do you have any difficulties with using the toilet, including getting up and down?
Continence	do you have any difficulties with controlling urination and defecation?
Housekeeping	do you have any difficulties with doing household chores?
Cooking	do you have any difficulties with preparing hot meals?
Shopping	do you have any difficulties with shopping for groceries?
Managing money	do you have any difficulties with managing your money,
	such as paying your bills, keeping track of expenses, or managing assets?
Taking medications	do you have any difficulties with taking medications?

*Note*: CHARLS wave 2011, 2013, 2015 and 2018.

Participants were asked whether they had difficulties for each activity because of health and memory problems. Responses range from 1) “I don’t have any difficulty” to 2) “I have difficulty but can still do it”, 3) “I have difficulty and need help” and 4) “I can not do it”. Given responses, participants with limitations in activities can be identified by either report of difficulty or report of need for help. In comparison with self-reported dependency, self-reported difficulty is less affected by social context like availability of family support [15, 27]. In this analysis, limitation in one activity of daily living is defined as report of difficulty or inability to perform such an activity. In contrast, no limitation is defined as report of no difficulty in performing such an activity.

### Analysis methods

This study starts with a descriptive analysis to explore the prevalence of activity limitation in ADL/IADL activities in the years 2011 and 2018. The incidence of limitation, which represents difference in prevalence over a seven-year interval, is calculated and its significance is assessed using a t-test. However, it is worth noticing that the incidence is subject to censoring and truncation due to death or late entry.

To deal with problems of censoring and truncation, the Kaplan-Meier estimate and Cox regression are used to study the transition from limitation-free status to limitation for each activity. Participants who hadn’t become limited by the last survey, have died or dropped out between surveys are censored. For instance, if a participant was healthy in 2011, remained healthy in 2015, but dropped out in 2018, their duration is considered as censoring at 2015. Participants who were older than 45 when entry into the survey is considered cases of left truncation. Note that distinct samples are used for the analysis of each activity. The median survival time is determined from the Kaplan-Meier product-limit estimate of the survivor function. Median survival time for both males and females are reported and their difference is evaluated using a bootstrapped t-test.

To gain further insights into the development of activity limitations, Cox regressions are employed to investigate the influence of health conditions and contextual factors on the onset of activity limitation. The study includes self-reported health and the number of chronic illnesses as measures of health condition, as well as personal factors such as sex, urban or rural residence, education, and marital status. Environmental factors, such as the availability of seated toilets, shower or bathing facilities, telephone, electricity, running water indoors, and elderly care centers in the community, are also considered.

Population sampling weights are applied with adjustments made for household and individual non-response. Analyses are conducted using Stata 16.1 (StataCorp, College Station, TX 77845, USA).

## Results

### Descriptive analysis

Table 2 presents the descriptive statistics of our sample. The average age of participants is approximately 60 years old. About 25% of participants report poor health, and on average, each participant has 1.37 chronic illnesses. The sample is evenly distributed between males and females, with roughly half of the participants residing in rural areas. The majority of participants (86%) are married. In terms of education, 41% have received education above elementary school but below high school, 15% have completed high school education, and 44% have only received education at the elementary school level or below.

**Table 2:** Descriptive statistics of the study sample.

	Year 2011	Year 2018	Diff
ADL/IADLactivitieslimitation(meanvalues)			
Dressing	0.05 (0.22)	0.08 (0.28)	0.04*** [0.00]
Bathing	0.07 (0.25)	0.10 (0.30)	0.06*** [0.00]
Feeding	0.03 (0.18)	0.03 (0.18)	0.01*** [0.00]
Transferring	0.06 (0.23)	0.08 (0.28)	0.04*** [0.00]
Toileting	0.11 (0.31)	0.13 (0.34)	0.04*** [0.00]
Continence	0.04 (0.20)	0.06 (0.23)	0.03*** [0.00]
Housekeeping	0.10 (0.30)	0.17 (0.38)	0.10*** [0.00]
Cooking	0.09 (0.29)	0.13 (0.34)	0.07*** [0.00]
Shopping	0.09 (0.29)	0.11 (0.31)	0.04*** [0.00]
Managing money	0.12 (0.33)	0.13 (0.34)	0.03*** [0.00]
Taking medications	0.07 (0.25)	0.07 (0.25)	0.01*** [0.00]
Baselinecharacteristics(meanvalues)			
Age	59.76 (10.20)		
Poor SRH	0.25 (0.43)		
Number of chronic illnesses	1.37 (1.41)		
Female	0.52 (0.50)		
Rural area	0.50 (0.50)		
Below high school	0.41 (0.49)		
High school and above	0.15 (0.35)		
Being married	0.86 (0.35)		
Toilet with a seat	0.25 (0.43)		
Shower/bath facilities	0.45 (0.50)		
Telephone	0.50 (0.50)		
Electricity	0.85 (0.35)		
Running water	0.69 (0.46)		
Elderly care center	0.11 (0.31)		

*Note*: *N* = 16, 381. Statistics are weighted by population sampling weights. Prevalence ordering in 2011/2018: dressing (9/8), bathing (6/6), feeding (11/11), transferring (8/7), toileting (2/3), continence (10/10), housekeeping (3/1), cooking (4/2), shopping (5/5), managing money (1/4), taking medications (7/9). Standard deviations in parentheses and standard errors in brackets.

*p < 0.1,

**p < 0.05,

***p < 0.01

We find that there is a lack of disability-friendly living facilities among the participants. Only 25% of participants have access to toilets with seats. The availability rates for shower/bath facilities, telephone, running water, and care centers are also relatively low, at 45%, 50%, 85%, and 11% respectively. This finding confirms our hypothesis H1.

At baseline in 2011, the ordering of limitation prevalence is managing money (12%), toileting (11%), housekeeping (10%), shopping (9%), cooking (9%), bathing (7%), taking medications (7%), transferring (6%), dressing (5%), continence (4%) and feeding (3%). In 2018, the ordering becomes housekeeping (17%), cooking (13%), toileting (13%), managing money (13%), shopping (11%), bathing (10%), transferring (8%), dressing (8%), taking medications (7%), continence (6%) and feeding (3%). The incidence increase is largest for housekeeping (+10%), following by cooking (+7%), bathing (+6%), dressing (+4%), toileting (+4%), transferring (+4%), shopping (+4%), continence (+3%), managing money (+3%), feeding (+1%) and taking medications (+1%). It is worth noting that these changes are biased by sample attrition, primarily due to death, which would be addressed in the survival analyses. Nevertheless, the large difference in prevalence at a particular time and unequal change along time across items reveals a rather complicated progression pattern of limitation of different activities with age.

### Kaplan-Meier estimate

Table 3 shows the median age at onset of limitation for the whole population and for men and women separately. Half of the population become limited in activities including housekeeping, toileting and managing money in their early 70s, followed by activities including cooking, shopping, bathing and transferring in their late 70s and activities including dressing, taking medications and continence in their early 80s. Finally, half of the population become limited in feeding in their early 90s. Women show significant younger age of limitation onsets for all activities except of continence and feeding. There is a minimal sex difference in continence and the difference in feeding limitations is not statistically significant due to very low incidence.

**Table 3:** Median age in years of onset of limitations in ADL/IADL items.

ADL/IADL limitation	Median			Diff. Men-Women	*N*
	All	Women	Men		
Dressing	81.0 (68.0, 94.0)	79.0 (67.0, 91.0)	82.0 (70.0, †)	3.0*** [1.1,4.9]	15546
Bathing	79.0 (67.0, 89.0)	76.0 (65.0, 86.0)	81.0 (70.0, 92.0)	5.0*** [3.4,6.6]	15299
Feeding	92.0 (79.0, †)	91.0 (79.0, †)	93.0 (79.0, †)	2.0 [-1.4,5.4]	15906
Transferring	79.0 (67.0, 92.0)	74.0 (63.0, 88.0)	83.0 (72.0, †)	9.0*** [7.1,10.9]	15484
Toileting	71.0 (60.0, 82.0)	67.0 (58.0, 78.0)	75.0 (64.0, 87.0)	8.0*** [6.5,9.5]	14499
Continence	84.0 (72.0, 97.0)	84.0 (70.0, 94.0)	84.0 (73.0, †)	0.0 [-3.0,3.0]	15677
Housekeeping	70.0 (60.0, 80.0)	67.0 (58.0, 77.0)	74.0 (63.0, 83.0)	7.0*** [5.7,8.3]	14833
Cooking	75.0 (64.0, 84.0)	73.0 (63.0, 83.0)	76.0 (65.0, 86.0)	3.0*** [1.6,4.4]	14920
Shopping	76.0 (65.0, 85.0)	73.0 (62.0, 82.0)	80.0 (70.0, 90.0)	7.0*** [5.7,8.3]	14884
Managing money	71.0 (59.0, 84.0)	67.0 (57.0, 79.0)	76.0 (64.0, 88.0)	9.0*** [7.4,10.6]	14304
Taking medications	82.0 (68.0, 92.0)	78.0 (64.0, 90.0)	86.0 (74.0, †)	8.0*** [5.8,10.2]	15266

*Note*: Population sampling weights are applied. Interquartile range (25%, 75%) in parenthesis and 95% CI in bracket.

^†^ Nonestimable. *Diff* is defined as the difference of median age in years of onset of limitations between men and women.

* *p* < 0.1,

** *p* < 0.05,

*** *p* < 0.01.

**Fig 2:**
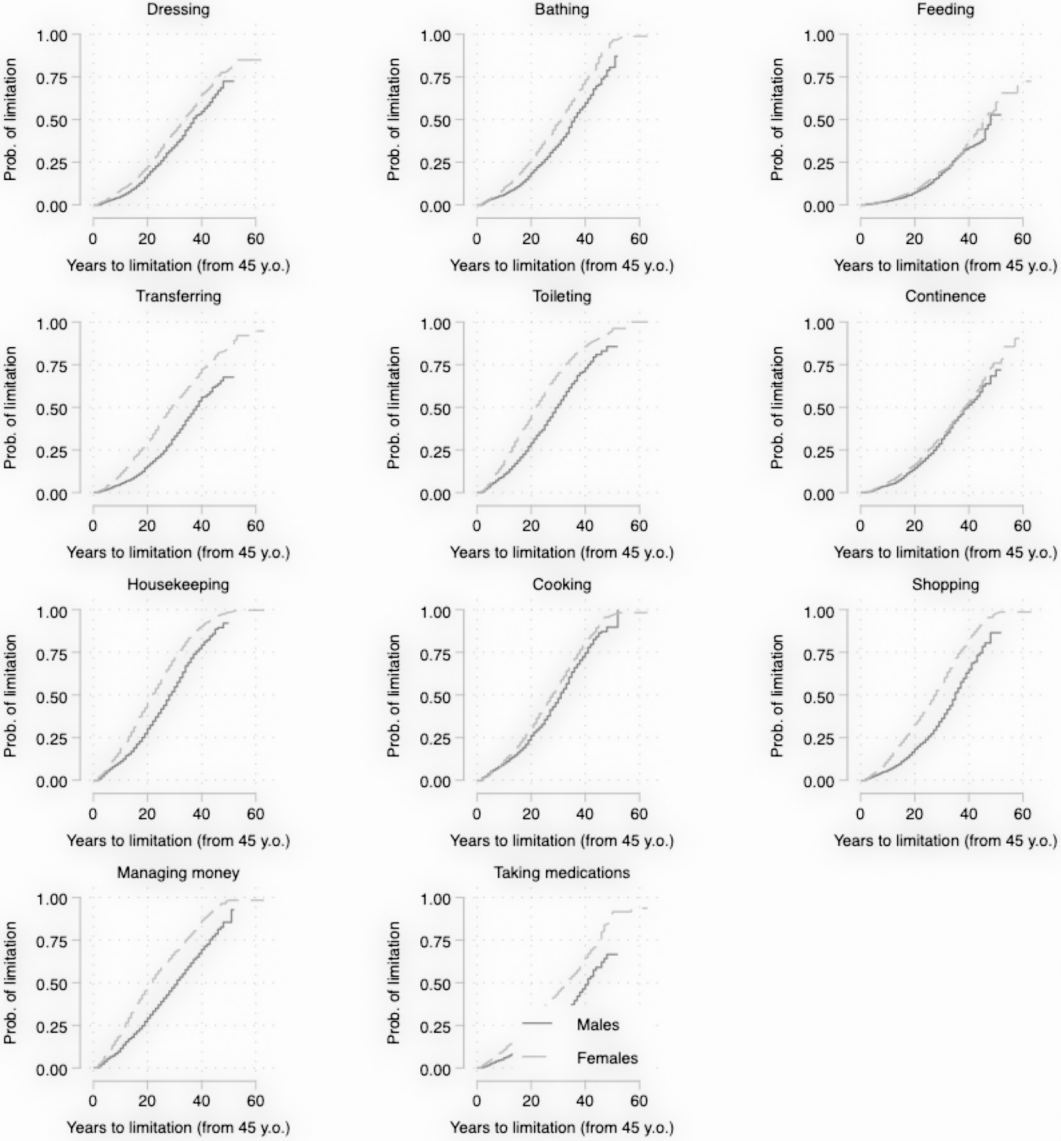
Age distribution of limitations in activities of daily living. Limitation in one activity of daily living is defined as report of difficulty or inability to perform such an activity, while no limitation is defined as report of no difficulty in performing such an activity. Probability curves are estimated using Kaplan Meier method. The origin time of analysis is set to be 45 years old, observations older than 110 years old are right-censored. Population sampling weights are applied.

Fig 2 presents the age distribution of the onset of limitation for each activity of daily living for females and males. Unsurprisingly the cumulative hazards for all activities increase with age, but there is no clear separation between ADL and IADL curves. Limitations in housekeeping, toileting and managing money tend to strike earlier in life than other activities. The curve for feeding is strongly dominated by other curves, implying the risk of feeding limitation is relatively low at all ages. Sex difference is obvious for all activities except feeding and continence.

### Cox regression

The results of the Cox regression analysis (Table 4) demonstrate the associations of various health conditions and contextual factors with the onset of activity limitation for ADL/IADL activities. Poor health is consistently linked to higher hazards of limitation across all activities, indicating that individuals in poor health are more likely to experience limitations in their daily activities. Similarly, a higher number of chronic illnesses is associated with increased hazards of activity limitation. Personal factors also play a significant role in activity limitation. Being males and having better education, is associated with reduced risk of limitation for most activities but continence. Among the environmental factors, the presence of a toilet with a seat, shower/bath facilities, and running water is found to be protective against activity limitations. Individuals with access to these amenities experience lower hazards of limitation for most activities. In particular, it is noteworthy that the absence of toilets with seats increases the hazards of limitation for all activities, particularly impacting the development of limitations in toileting. This finding suggests that the lack of toilet seats, combined with their low availability, may contribute to the early onset of toileting limitations among Chinese older adults. These findings provide supportive evidence for our hypotheses H2 and H3.

**Table 4:** Cox regression of ADL/IADL activity limitation on health condition and contextual factors.

	Dressing	Bathing	Feeding	Transferring	Toileting	Continence	Housekeeping	Cooking	Shopping	Managing money	Taking medications
Poor SRH	2.0*** (0.1)	2.0*** (0.1)	2.0*** (0.2)	1.9*** (0.1)	1.6*** (0.1)	2.0*** (0.1)	1.9***(0.1)	1.9*** (0.1)	1.7*** (0.1)	1.6*** (0.1)	1.8*** (0.1)
Number of chronic illnesses	1.1*** (0.0)	1.2*** (0.0)	1.1*** (0.0)	1.2*** (0.0)	1.2*** (0.0)	1.2*** (0.0)	1.2*** (0.0)	1.1*** (0.0)	1.1*** (0.0)	1.1*** (0.0)	1.1*** (0.0)
Female	1.1 (0.1)	1.2*** (0.1)	0.9 (0.1)	1.5*** (0.1)	1.4*** (0.1)	1.0 (0.1)	1.4*** (0.1)	1.1** (0.0)	1.5*** (0.1)	1.4*** (0.1)	1.4*** (0.1)
Rural area	1.1* (0.1)	0.9 (0.1)	1.1 (0.1)	1.3*** (0.1)	1.0 (0.0)	1.1 (0.1)	1.1 (0.0)	1.1 (0.1)	1.1 (0.1)	1.1. (0.1)	1.0 (0.1)
Below high school	0.8*** (0.0)	0.8*** (0.0)	0.7*** (0.1)	0.7*** (0.0)	0.8*** (0.0)	0.9 (0.1)	0.8*** (0.0)	0.8*** (0.0)	0.7*** (0.0)	0.6*** (0.0)	0.6*** (0.0)
High school and above	0.5*** (0.1)	0.6*** (0.1)	0.5*** (0.1)	0.5*** (0.1)	0.7*** (0.1)	0.9 (0.1)	0.6*** (0.1)	0.6*** (0.1)	0.4*** (0.1)	0.3*** (0.0)	0.4*** (0.1)
Being married	1.0 (0.1)	0.9 (0.1)	1.0 (0.1)	1.0 (0.1)	0.9** (0.0)	1.0 (0.1)	1.0 (0.0)	1.1 (0.1)	1.1** (0.1)	1.1. (0.1)	1.0 (0.1)
Toilet with a seat	0.7*** (0.1)	0.8*** (0.1)	0.8* (0.1)	0.8** (0.1)	0.6*** (0.0)	0.8*** (0.1)	0.8*** (0.1)	0.8*** (0.1)	0.9* (0.1)	0.7*** (0.1)	0.8*** (0.1)
Shower/bath facilities	0.8*** (0.1)	0.7*** (0.0)	0.7*** (0.1)	0.8*** (0.0)	0.9* (0.0)	0.9** (0.1)	0.9*** (0.0)	0.8*** (0.0)	0.8*** (0.0)	0.9***	0.9** (0.1)
Telephone	0.9 (0.0)	0.9 (0.0)	0.8*** (0.1)	0.9 (0.0)	1.0 (0.0)	0.9* (0.1)	1.0 (0.0)	1.0 (0.0)	0.9** (0.0)	0.9** (0.0)	0.9** (0.0)
Electricity	1.0 (0.1)	0.9 (0.1)	0.9 (0.1)	1.0 (0.1)	1.0 (0.1)	1.1 (0.1)	1.0 (0.0)	0.9 (0.1)	0.9* (0.1)	0.9** (0.0)	1.1 (0.1)
Running water	0.9 (0.1)	0.9*** (0.0)	1.0 (0.1)	0.9 (0.0)	0.9* (0.0)	0.8*** (0.1)	0.9* (0.0)	0.9** (0.0)	0.9*** (0.0)	0.9* (0.0)	0.8*** (0.1)
Elderly care center	0.9 (0.1)	0.8 (0.1)	1.0 (0.2)	0.9 (0.1)	0.9 (0.1)	1.0 (0.1)	0.9 (0.1)	0.8* (0.1)	0.8 (0.1)	0.8** (0.1)	0.8 (0.1)
N	15546	15299	15906	15484	14499	15677	14833	14920	14884	14304	15266

*Note*: Standard errors in parenthesis.

*p < 0.1,

**p < 0.05,

***p < 0.01.

## Discussion

This study provides new evidence on patterns of activity limitation among middle-aged and older adults in China using a nationally representative sample and a longitudinal design over seven years (from 2011 to 2018).

Concerning ADL items, people first lose the ability of toileting, followed by bathing, transferring, dressing, continence, and finally, feeding. Our results are broadly consistent with early studies on ADL hierarchy, finding that older adults tend to first become limited in activities that require the strength of the lower extremity before activities that require the strength of the upper extremity [16, 21]. However, we find that toileting strikes people much earlier than in other studies. This early onset of limitation on toileting is likely due to widely equipped squat toilets in China that may require more strength of lower extremities and thus be more challenging for older adults. Using data from surveys of the oldest old, one study finds that toileting is among the first activities restricted for Chinese [26], and another study finds that both older Americans and Chinese have difficulty with bathing first and eating last, but for Chinese toileting become challenging soon after bathing [17].

Our results reveal that ADL and IADL items do not necessarily follow an IADL-first-ADL-second hierarchy. While most IADL items develop earlier than ADL items, the development does not follow a strict hierarchy. Overlapping of ADL and IADL losses are also reported in previous studies [19, 23]. Toileting limitation onsets earlier than most IADL items and taking medicine is limitation-free until the age of losing continence control.

Against most existing studies focusing on the oldest-old adults, our results show that the onsets of limitations in activity limitations take place much earlier in China. More than 25% of people already have difficulty in all activities except continence and feeding as early as 70 years old. By 80 years, more than half of people become limited in all activities but vital ones like dressing, feeding, continence, and taking medicines. The early onsets of limitations are likely attributable to a rising prevalence of chronic illnesses, lack of facilities, and a range of failures in the healthcare system, especially in primary care [28–30]. A further inquiry into the mechanism of early onsets of functional limitations could be a direction for future research.

While previous studies have found that women have an earlier onset of limitation than men in ADL activities [15], we have shown that the sex gap exists not only for ADLs but also for IADLs. Our result is consistent with studies that have found that women have higher odds than men of becoming limited in ADL and IADL activities [18, 23]. Furthermore, our finding is generally in line with the literature on the male-female health-survival paradox, i.e., women outlive men but are less healthier than men [31].

When studying limitation patterns in activities of daily living, previous studies often draw the sample from the oldest-old, for example, older adults aged 75 and above. One study based on a UK population investigates the pattern of ADL limitations [15]. However, such a sample, comprising limitation-free people at the age of entry (for example, 75), is rather selective and biased towards a relatively healthy population. In contrast, our analysis includes not the oldest-old, but also the middle-aged and young-old aged 45 and above, enabling us to trace the onset of limitation for a general population.

Nevertheless, to compare our results with previous studies and gain further insights, we decided to refine our analysis by focusing on a specific subgroup of the population. Therefore, we reanalyzed the data by restricting our analysis to a sample composed only of adults aged 75 and above. This age group is of particular interest as it represents the segment of the population most likely to experience age-related limitations in daily activities.

The findings from this subgroup analysis are presented in Table 5, which displays the median age of onset for basic activities of daily living (ADL). Interestingly, the observed pattern aligns closely with the findings from a study conducted in the UK [15]. In both studies, it was found that older adults tend to experience limitations first in activities such as bathing and toileting, followed by dressing and transferring, and finally feeding. This consistent pattern suggests a universal trend in the development of activity limitations among older adults.

**Table 5:** Median age in years of onset of limitations in ADL items for those aged 75 and older.

ADL limitation	Median (interquartile range)	
	China	UK ⋆
Dressing	90.0 (83.0, †)	92.2 (87.2, 97.2)
Bathing	87.0 (81.0, 92.0)	81.5 (78.7, 85.4)
Feeding	97.0 (88.0, †)	† (95.7, †)
Transferring	90.0 (83.0, 98.0)	95.5 (90.2, †)
Toileting	86.0 (80.0, 94.0)	91.7 (85.3, 95.5)
Continence	92.0 (84.0, 98.0)	‡

*Note*: *N* = 2, 990. Population sampling weights are applied. Interquartile range (25%, 75%) in parenthesis.

^⋆^ Estimates based on a UK population [15].

^†^ Nonestimable.

^‡^ Not included in the survey.

Ordering by median age in China/UK (by first quartile if median age is missing or the same): dressing (3/3), bathing (2/1), feeding (5/5), transferring (4/4) and toileting (1/2).

However, there are notable differences between the Chinese older adult population in our study and their counterparts in the UK. Firstly, Chinese older adults tend to experience limitations at an earlier age compared to their UK counterparts for all activities except bathing, which supports our hypothesis H4. The lower risk of bathing limitation among Chinese older adults may be attributed to cultural factors, such as a lower frequency of bathing or a higher preference for using showers instead of tubs, which require less physical effort.

Secondly, there is a distinct difference in the order of limitations between bathing and toileting for the two populations. In our sample, we observed a reversed order, with toileting limitations preceding bathing limitations. This finding can be attributed to the fact that a significant proportion of the toilets accessible to older adults in our study lacked seats. As individuals age, the absence of a seat on the toilet can pose increased difficulty and discomfort, leading to earlier onset of toileting limitations. This highlights the importance of environmental factors and the need for age-friendly facilities and infrastructure to support the functional independence of older adults.

We acknowledge the limitations of our study. Firstly, while our study relies on a longitudinal design which covers a relatively long period from 2011 to 2018, we cannot capture each individual’s complete development of functional limitations. Our sample might be selective at baseline and not immune from survival bias. For example, men with early onset of disability might have worse survival so they are less likely to be included in the baseline survey. If that is case, our sample tend to include men with better health, and we would observe the pattern that men develop limitation later than women. The bias might be large for activities developed later in the life course, such as feeding.

Secondly, as a survey representing the population of middle-aged and older people in China, CHARLS has a relatively small cohort for the oldest-old. Consequently, our estimates of certain limitations, such as feeding, mainly developed at a very late stage of life, can be subject to considerable variance.

Thirdly, it is important to acknowledge that our study treat censoring due to institutionalization and dropping-out of the survey as uninformative. Similarly, competing events like death are considered as uninformative censoring. This assumption justifies the use of Kaplan-Meier and Cox methods. However, it is worth noting that death and institutionalization may actually be informative and correlated with the risks of activity limitation. This could introduce bias in our estimates when utilizing both Kaplan-Meier and Cox methods.

Fourthly, it is necessary to consider the potential bias in estimates of the prevalence of disability, as our measures of activity limitation are based on self-reporting. Different individuals may use varying response scales, leading to potential reporting bias in our estimates. One potential solution to address this issue is the use of anchoring vignettes, which can help correct for reporting bias. Unfortunately, the current survey did not include anchoring vignettes specifically related to ADL/IADL activities.

Lastly, in the survival analysis, we have estimated each limitation’s unconditional distribution of age. Though it would be informative to model the joint distribution of all these eleven ADL/IADL items, the estimation task is daunting because of the curse of dimensionality. We, therefore, decide to leave the inquiry to future research.

Our study is informative in measuring the functional ability of older adults and decision-making of long-term care policies and clinical interventions in China and elsewhere. First, it is beneficial to include more items than ADL as an inventory of assessment of disability. Second, the prevalence estimates based on a nationally representative sample are helpful for policy-makers in planning long-term care. Third, onset timing and order aid the clinician in identifying and monitoring older adults with high risks. Fourth, our results show that limitation of ADL activities takes much earlier in China than in older adults in the UK, which calls for a national plan to prevent early disability. Last, the gender gap in disability should be addressed in policies on public health and aging.

## Conclusion

The measuring onset of activity limitation potentially helps policymakers and medical practitioners understand how these limitations unfold asynchronously with aging and prepare to take action in time. Early onset of functional activity limitations in Chinese older adults and the age difference of onset between men and women should be addressed when drafting public policies on long-term care. Personal and environmental factors should be targeted to prevent early onset of limitation.

## Supporting information

S1 Appendix (PDF)

S1 Dataset (ZIP)
