# Supplementary material for: Onset of ADL and IADL limitation among Chinese middle-aged and older adults
Source: PLoS One. 2023 Jul 17;18(7):e0287856. doi: 10.1371/journal.pone.0287856 (PMC10351716; doi:10.1371/journal.pone.0287856)
Supplement: S1 Appendix — (PDF) [file pone.0287856.s001.pdf]

Onset of ADL and IADL Limitation Among Chinese Middle-aged and Older Adults - Appendices

Wenyuan Zheng[1], Zhiyong Huang[2*]

1 Department of Insurance, Southwestern University of Finance and Economics, Chengdu, China
2 Department of Social Security, Southwestern University of Finance and Economics, Chengdu, China

* zhiyonghuang@swufe.edu.cn

Table 1. Life table for limitation in dressing

	Start time	End time	Beginning total	Events during interval	Lost	Survival	Failure
1	45	50	15546	29	255	1	0.00
2	50	55	15262	137	1804	1	0.01
3	55	60	13321	200	2634	1	0.03
4	60	65	10487	306	2825	1	0.06
5	65	70	7356	327	2474	1	0.11
6	70	75	4555	273	1662	1	0.18
7	75	80	2620	208	1132	1	0.26
8	80	85	1280	129	653	1	0.36
9	85	90	498	58	313	1	0.47
10	90	95	127	18	79	0	0.58
11	95	100	30	3	24	0	0.65
12	100	105	3	0	2	0	0.65
13	105	110	1	0	1	0	0.65

Table 2. Life table for limitation in dressing

	Start time	End time	Beginning total	Events during interval	Lost	Survival	Failure
1	45	50	15299	42	254	1	0.00
2	50	55	15003	140	1777	1	0.01
3	55	60	13086	232	2612	1	0.03
4	60	65	10242	332	2759	1	0.07
5	65	70	7151	347	2426	1	0.12
6	70	75	4378	278	1599	1	0.19
7	75	80	2501	238	1072	1	0.29
8	80	85	1191	165	583	1	0.42
9	85	90	443	81	260	0	0.57
10	90	95	102	37	47	0	0.77
11	95	100	18	5	9	0	0.86
12	100	105	4	1	2	0	0.90
13	105	110	1	0	1	0	0.90

Table 3. Life table for limitation in dressing

	Start time	End time	Beginning total	Events during interval	Lost	Survival	Failure
1	45	50	15906	9	261	1	0.00
2	50	55	15636	49	1878	1	0.00
3	55	60	13709	77	2767	1	0.01
4	60	65	10865	122	3042	1	0.02
5	65	70	7701	153	2750	1	0.05
6	70	75	4798	145	1835	1	0.08
7	75	80	2818	117	1303	1	0.13
8	80	85	1398	80	766	1	0.20
9	85	90	552	30	376	1	0.27
10	90	95	146	19	93	1	0.41
11	95	100	34	4	24	0	0.51
12	100	105	6	1	3	0	0.62
13	105	110	2	0	2	0	0.62

Table 4. Life table for limitation in dressing

	Start time	End time	Beginning total	Events during interval	Lost	Survival	Failure
1	45	50	15484	30	258	1	0.00
2	50	55	15196	166	1786	1	0.01
3	55	60	13244	249	2592	1	0.03
4	60	65	10403	348	2786	1	0.07
5	65	70	7269	334	2459	1	0.12
6	70	75	4476	306	1622	1	0.20
7	75	80	2548	213	1106	1	0.28
8	80	85	1229	136	629	1	0.39
9	85	90	464	56	288	1	0.50
10	90	95	120	16	79	0	0.60
11	95	100	25	5	16	0	0.71
12	100	105	4	1	2	0	0.81
13	105	110	1	0	1	0	0.81

Table 5. Life table for limitation in dressing

	Start time	End time	Beginning total	Events during interval	Lost	Survival	Failure
1	45	50	14499	67	250	1	0.00
2	50	55	14182	276	1680	1	0.03
3	55	60	12226	419	2367	1	0.06
4	60	65	9440	523	2440	1	0.12
5	65	70	6477	494	2085	1	0.20
6	70	75	3898	402	1312	1	0.30
7	75	80	2184	286	876	1	0.42
8	80	85	1022	157	488	0	0.53
9	85	90	377	64	228	0	0.65
10	90	95	85	20	46	0	0.76
11	95	100	19	3	13	0	0.82
12	100	105	3	1	1	0	0.89
13	105	110	1	0	1	0	0.89

Table 6. Life table for limitation in dressing

	Start time	End time	Beginning total	Events during interval	Lost	Survival	Failure
1	45	50	15677	19	262	1	0.00
2	50	55	15396	113	1827	1	0.01
3	55	60	13456	151	2679	1	0.02
4	60	65	10626	240	2914	1	0.05
5	65	70	7472	259	2567	1	0.09
6	70	75	4646	214	1737	1	0.14
7	75	80	2695	199	1176	1	0.22
8	80	85	1320	112	707	1	0.31
9	85	90	501	51	326	1	0.41
10	90	95	124	23	76	0	0.57
11	95	100	25	3	17	0	0.65
12	100	105	5	1	3	0	0.75
13	105	110	1	0	1	0	0.75

Table 7. Life table for limitation in dressing

	Start time	End time	Beginning total	Events during interval	Lost	Survival	Failure
1	45	50	14833	72	240	1	0.00
2	50	55	14521	278	1678	1	0.03
3	55	60	12565	385	2415	1	0.06
4	60	65	9765	590	2467	1	0.12
5	65	70	6708	571	2139	1	0.21
6	70	75	3998	464	1321	1	0.32
7	75	80	2213	388	842	1	0.47
8	80	85	983	208	442	0	0.61
9	85	90	333	84	180	0	0.75
10	90	95	69	27	30	0	0.87
11	95	100	12	4	6	0	0.93
12	100	105	2	1	0	0	0.96
13	105	110	1	0	1	0	0.96

Table 8. Life table for limitation in dressing

	Start time	End time	Beginning total	Events during interval	Lost	Survival	Failure
1	45	50	14920	47	252	1	0.00
2	50	55	14621	199	1739	1	0.02
3	55	60	12683	272	2511	1	0.04
4	60	65	9900	426	2647	1	0.09
5	65	70	6827	418	2319	1	0.16
6	70	75	4090	361	1458	1	0.25
7	75	80	2271	326	911	1	0.38
8	80	85	1034	191	494	0	0.53
9	85	90	349	90	187	0	0.70
10	90	95	72	29	31	0	0.85
11	95	100	12	4	6	0	0.92
12	100	105	2	0	1	0	0.92
13	105	110	1	0	1	0	0.92

Table 9. Life table for limitation in dressing

	Start time	End time	Beginning total	Events during interval	Lost	Survival	Failure
1	45	50	14884	44	253	1	0.00
2	50	55	14587	167	1752	1	0.02
3	55	60	12668	265	2544	1	0.04
4	60	65	9859	391	2643	1	0.08
5	65	70	6825	332	2346	1	0.14
6	70	75	4147	324	1529	1	0.22
7	75	80	2294	299	946	1	0.35
8	80	85	1049	187	509	0	0.50
9	85	90	353	83	199	0	0.66
10	90	95	71	28	33	0	0.84
11	95	100	10	2	7	0	0.89
12	105	110	1	0	1	0	0.89

Table 10. Life table for limitation in dressing

	Start time	End time	Beginning total	Events during interval	Lost	Survival	Failure
1	45	50	14304	84	239	1	0.01
2	50	55	13981	284	1635	1	0.03
3	55	60	12062	425	2311	1	0.07
4	60	65	9326	518	2394	1	0.12
5	65	70	6414	453	2053	1	0.20
6	70	75	3908	313	1421	1	0.28
7	75	80	2174	248	917	1	0.38
8	80	85	1009	156	498	0	0.51
9	85	90	355	80	207	0	0.66
10	90	95	68	24	32	0	0.82
11	95	100	12	2	8	0	0.86
12	100	105	2	0	1	0	0.86
13	105	110	1	0	1	0	0.86

Table 11. Life table for limitation in dressing

	Start time	End time	Beginning total	Events during interval	Lost	Survival	Failure
1	45	50	15266	44	255	1	0.00
2	50	55	14967	147	1768	1	0.01
3	55	60	13052	208	2560	1	0.03
4	60	65	10284	290	2786	1	0.06
5	65	70	7208	254	2483	1	0.10
6	70	75	4471	206	1687	1	0.15
7	75	80	2578	177	1144	1	0.23
8	80	85	1257	128	664	1	0.33
9	85	90	465	65	292	1	0.47
10	90	95	108	25	64	0	0.64
11	95	100	19	2	13	0	0.70
12	100	105	4	1	1	0	0.79
13	105	110	2	0	2	0	0.79
